# Advanced Applications of and Mechanistic Insights into Carbon-Based Nanomaterials in Agri-Food Safety Detection and Ecological Remediation

**DOI:** 10.3390/nano16150910

**Published:** 2026-07-24

**Authors:** Mei Wang, Jing Bai, Wei Lu, Bingliang Zhou, Xianghai Song, Quan Bu

**Affiliations:** 1 School of Agricultural Engineering, Jiangsu University, Zhenjiang 212013, Chinaqbu@ujs.edu.cn (Q.B.); 2 Institute of the Green Chemistry and Chemical Technology, School of Chemistry and Chemical Engineering, Jiangsu University, Zhenjiang 212013, China

**Keywords:** carbon-based nanomaterials, agri-food safety detection, ecological remediation, sensing mechanism, pollutant remediation

## Abstract

Pesticide and veterinary drug residues, heavy metals and other hazardous contaminants in agricultural products and food systems pose severe threats to food safety and agro-ecological security. Conventional detection techniques are plagued by complicated operations, long testing cycles and insufficient sensitivity, which fail to meet the practical requirements for rapid, accurate on-site detection and in situ remediation. This paper systematically introduces the fundamental physicochemical properties of typical carbon-based nanomaterials, including graphene, carbon nanotubes, carbon quantum dots and biomass-derived carbon. It comprehensively reviews the latest research advances of these materials in the detection of heavy metal ions, pesticide residues, mycotoxins and illegal additives, as well as in the non-destructive monitoring of food quality. Meanwhile, relevant applications of carbon-based nanomaterials in the adsorption, enrichment and catalytic remediation of heavy metals and organic pollutants in farmland soil and water environments are summarized. The intrinsic mechanisms underlying their performance in high-precision detection and environmental remediation are elaborated from the perspectives of optical sensing response and adsorption–separation effects. Furthermore, the current technical limitations and bottlenecks restricting the practical application of carbon-based nanomaterials are discussed. Combined with the industrial demands for rapid screening of agro-food safety risks and in situ treatment of farmland environments, the future development prospects of carbon-based nanomaterials in agriculture and food safety fields are outlined. This work aims to provide theoretical references for the development and industrialization of high-performance carbon-based sensing and remediation materials, and to facilitate the risk prevention and control of agro-food safety as well as the green and sustainable development of agricultural ecosystems.

## 1. Introduction

Against the backdrop of rapid advancement in global agricultural modernization, modern agricultural practices have greatly improved the production capacity and supply efficiency of grain, oil crops, fruits, vegetables, livestock and aquatic products [1]. Nevertheless, to pursue high and stable yields as well as pest and disease control in intensive agricultural production, chemical fertilizers and pesticides have been excessively applied over a long period. This has frequently led to excessive residues on the surface and inside the tissues of agricultural products [2]. Pesticide residues and fertilizer derivatives can accumulate in the human body continuously through dietary intake. Long-term exposure may damage the nervous system, metabolic system and immune function, posing a persistent potential threat to public health [3]. Along with industrialization, the indiscriminate discharge of industrial wastewater and the irrigation of farmland with substandard sewage in some agricultural areas have caused excessive levels of heavy metals such as cadmium and lead in cultivated soil and irrigation water [4]. Characterized by poor biodegradability, high bioaccumulation and irreversible hazards, heavy metals accumulate and amplify stepwise along the food chain before entering the human circulatory system. Their long-term accumulation can trigger severe health problems including carcinogenicity, teratogenicity and mutagenicity [5]. In addition, improper grain storage conditions, such as inappropriate temperature and humidity, poor ventilation and prolonged storage cycles, create favorable environments for mold growth and reproduction, which readily produce highly toxic mycotoxins represented by aflatoxins [6,7]. In livestock and aquaculture industries, problems including excessive breeding density, inadequate water environment management and insufficient disinfection easily cause massive proliferation of pathogenic bacteria such as Salmonella, resulting in microbial contamination of livestock and aquatic products and further triggering mass foodborne diseases [8]. Under such circumstances, establishing a full-process and multi-dimensional detection and monitoring system covering production environment, field cultivation, post-harvest storage and transportation, and end products has become a core solution to food safety governance. It also serves as an important guarantee for complying with international trade regulations on agricultural products, circumventing cross-border safety trade barriers, and promoting the high-quality and sustainable development of modern agriculture [9,10].

In the field of food and agricultural safety detection, four mainstream conventional analytical methods are widely adopted, namely chromatography, chromatography–mass spectrometry (MS), immunoassay and capillary electrophoresis (CE) [11]. Chromatographic techniques represented by high-performance liquid chromatography (HPLC) and gas chromatography (GC) exhibit excellent component separation capability and enable accurate quantitative detection of contaminants. However, they generally suffer from long analysis times and a high cost of testing equipment [12,13]. Chromatography–MS techniques such as liquid chromatography–MS (LC-MS and GC-MS) combine the superior separation performance of chromatography and the precise qualitative ability of MS, achieving extremely high detection sensitivity and accuracy. Even so, the high procurement and maintenance costs of relevant instruments, together with strict requirements for professional skills of operators, limit their application in on-site rapid detection [14,15]. Enzyme-linked immunosorbent assay (ELISA), a typical immunoassay, features simple operation and satisfactory detection specificity, and is suitable for high-throughput preliminary screening of large batches of samples. Its performance, nevertheless, is vulnerable to antibody quality, and non-specific cross-reactions remain an inherent defect [16]. CE can realize efficient separation with microscale samples, but it suffers from poor detection repeatability and also relies on large-scale precision instruments [17]. Overall, traditional detection methods are generally hampered by cumbersome sample pretreatment and low detection efficiency, which fail to meet the current demands for large-scale, real-time and on-line rapid detection in food and agriculture. This has greatly driven the research and development and practical application of novel rapid sensing technologies [18].

To address the bottlenecks of conventional detection techniques, novel nanomaterial-based detection technologies have emerged, bringing revolutionary progress to food and agricultural safety analysis [19]. Common nanomaterials include AuNPs, quantum dots, graphene and nanozymes. Owing to their unique physicochemical properties, these materials present outstanding sensing performance [20,21]. Carbon-based nanomaterials stand out with the most comprehensive performance, featuring four core advantages. First, they deliver ultrahigh sensing sensitivity. Thanks to their large specific surface area and high electron transport efficiency, these materials can identify trace pollutants [22,23]. For instance, the limit of detection of multi-walled carbon nanotubes for kanamycin in milk is as low as 1.12 × 10^−8^ mg/g [24], and that of nitrogen-doped graphene fluorescent sensors for amaranth in candy samples reaches 1.5 × 10^−7^ mg/g [25], which makes up for the low sensitivity of conventional detection methods. Second, they allow facile surface modification. Functional groups such as -COOH and -NH_2_ can be grafted onto the material surface [26] to stably immobilize biorecognition elements including enzymes, antibodies and aptamers [27], enabling specific recognition of targets and improving detection selectivity. Third, they possess intrinsic optical properties and nanozyme activity. Carbon quantum dots (CQDs) exhibit good biocompatibility and stable fluorescence, while carbon-based composite nanomaterials show peroxidase-like catalytic activity [28,29], which supports the construction of visual colorimetric sensing platforms. Fourth, they are adaptable to diverse application scenarios and compatible with multiple sensing modes such as electrochemistry, fluorescence [30,31], colorimetry and SERS. In electrochemical sensing, carbon nanomaterials are commonly used as electrode modifiers. By optimizing the interfacial electron transfer efficiency, the fabricated sensors can accurately detect lead ions (Pb^2+^) [32], pesticide residues [33] and mycotoxins [34] in food matrices. In addition, these materials can serve as solid-phase adsorbents for the QuEChERS method to pretreat samples from complex food matrices and achieve simultaneous detection of multiple components when combined with chromatography–mass spectrometry [35].

**Figure 1:**
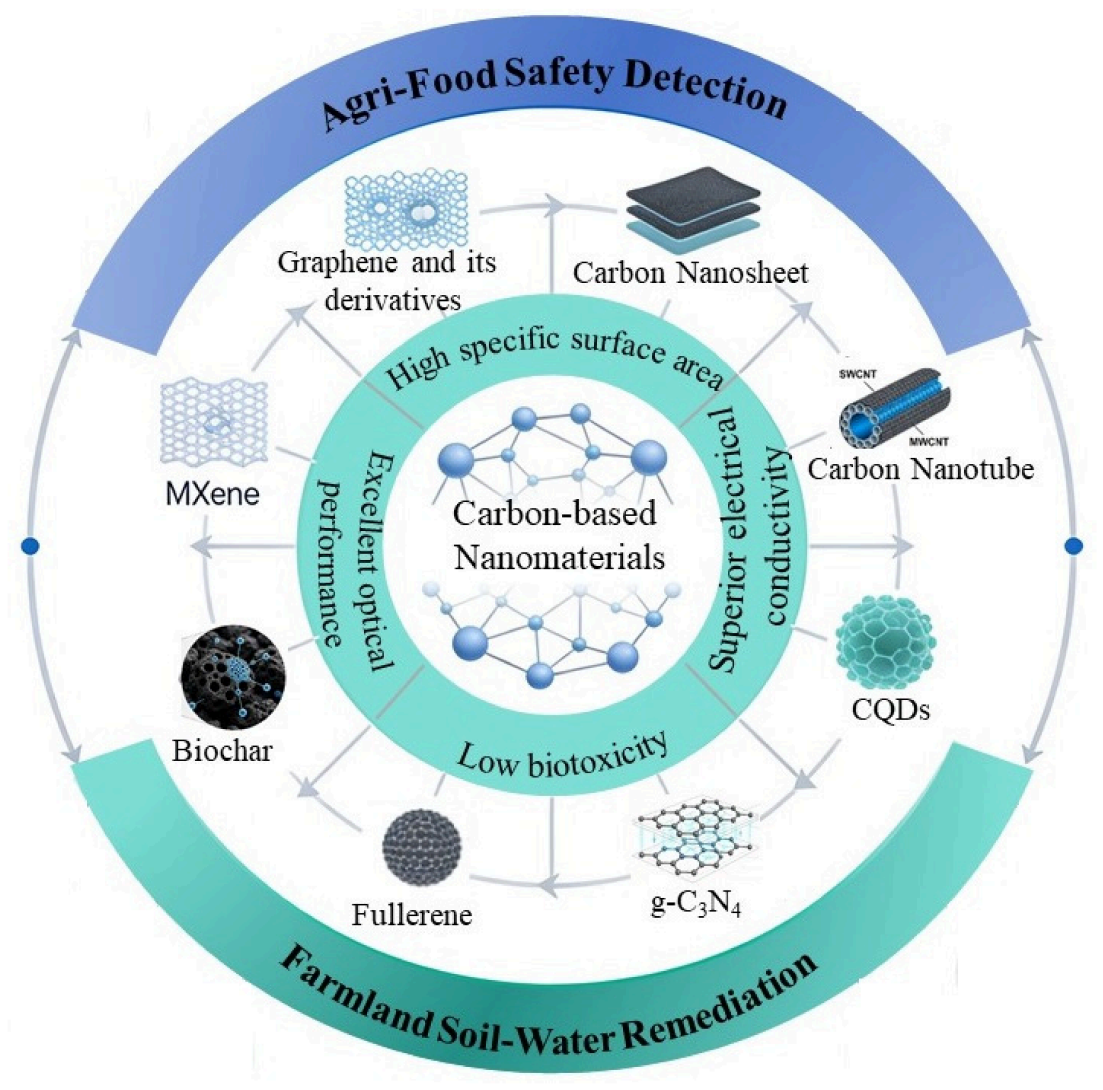
Properties and applications of carbon-based nanomaterials in agri-food safety detection and farmland remediation.

Combined with the four core advantages of carbon-based nanomaterials summarized above, Figure 1 systematically sorts out all core carbon-based materials investigated in this work as well as their two major application categories. It covers seven representative carbon-based nanomaterials including graphene, carbon nanotubes, carbon quantum dots, biochar, graphitic carbon nitride (g-C_3_N_4_), MXene and fullerenes, which span two-dimensional, one-dimensional, zero-dimensional and novel layered carbon materials, fully supporting the core argument of this review that carbon-based nanomaterials feature diverse structures and can be applied to various agricultural and food safety scenarios. The circular framework is divided into two functional modules: agri-food safety detection and farmland soil water remediation. The inner ring summarizes the intrinsic shared properties of carbon-based nanomaterials, namely high specific surface area, superior electrical conductivity, favorable optical performance and low biotoxicity, which visually elucidate the inherent advantages of such materials for sensing detection and environmental adsorption remediation. Two key conclusions can be drawn from this figure: first, carbon-based nanomaterials with distinct dimensions and configurations share favorable physicochemical properties, making them versatile media for agricultural and food safety applications; second, carbon-based nanomaterials form an integrated system of “accurate contaminant detection and in situ soil water pollution remediation”. They are capable of highly sensitive identification of trace contaminants in food matrices as well as adsorptive removal of pollutants in farmland soil and water, highlighting the broad application potential of carbon-based nanomaterials across the full industrial chain of agricultural safety from a macroscopic perspective.

**Figure 2:**
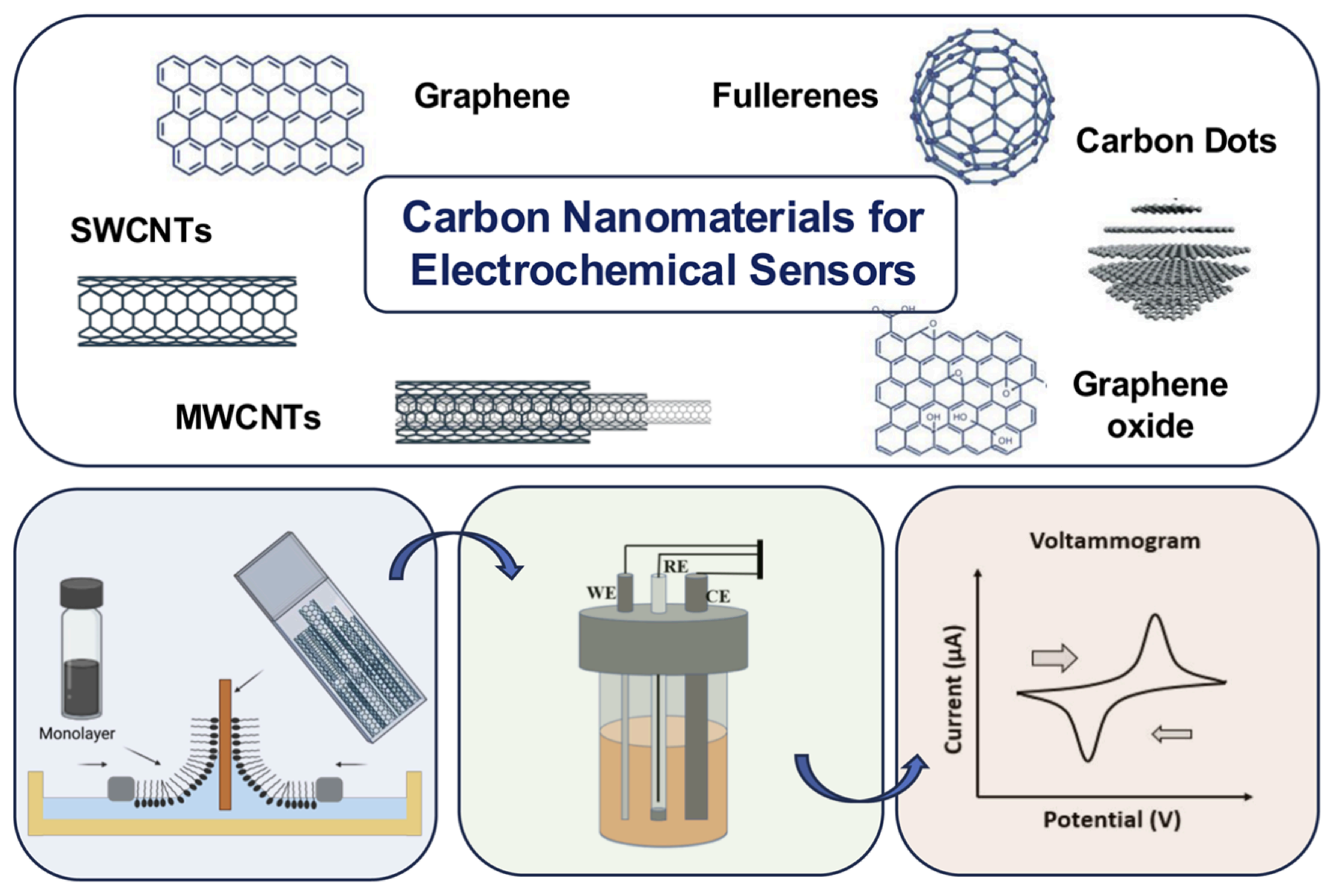
Carbon nanomaterial-based electrochemical sensor for multi-analyte detection with high sensitivity and selectivity [36].

Relying on the above intrinsic physicochemical advantages, carbon-based nanomaterials can be employed to construct integrated multimodal sensing platforms. In electrochemical detection systems, carbon-based substances are frequently adopted as electrode modification media to markedly accelerate interfacial electron transfer, enabling accurate quantitative analysis of diverse chemicals, veterinary drugs, biomarkers, environmental contaminants and gaseous analytes, which endows them with promising industrialization prospects (Figure 2) [36]. Within the full multimodal sensing framework built by carbon-based nanomaterials, electrochemical sensing stands out as the dominant analytical technique for quantifying veterinary and pesticide residues, heavy metal ions and various hazardous pollutants in agricultural food matrices owing to its stable signal output and ultrahigh detection sensitivity. Accordingly, Figure 2 collects a series of classic electrode-modified carbon materials including graphene, single/multi-walled carbon nanotubes, carbon dots and fullerenes, and intuitively illustrates the complete fabrication and detection workflow of carbon-based electrochemical sensors from substrate modification to signal readout. Apart from the electrochemical route, fluorescence sensing adopts carbon quantum dots and graphene quantum dots as optical probes to realize rapid identification of trace pollutants via fluorescence resonance energy transfer (FRET) and photoinduced electron transfer (PET) effects. Colorimetric sensing systems leverage the enzyme-mimetic catalytic activity of carbon-based nanozymes to trigger chromogenic reactions for direct visual on-site screening. Meanwhile, modified magnetic carbon-based composites can efficiently enrich and purify complex samples to greatly mitigate matrix effects, making them compatible with high-throughput batch analysis by large-scale chromatography–mass spectrometry instruments. All the above carbon-based sensing platforms feature ultra-fast responses, cutting the analysis cycle of traditional detection methods from several days down to mere minutes. They lay critical foundations for the development of low-cost rapid test strips and portable analytical devices, and are widely applicable to on-site screening scenarios such as farmlands and fresh food markets [37,38,39].

This paper systematically reviews the latest research advances of carbon-based nanomaterials, including graphene, CNTs, CQDs, graphitic carbon nitride (g-C_3_N_4_) and MXenes, in agricultural and food safety detection. We mainly summarize their applications in constructing various sensors, such as electrochemical, fluorescent, colorimetric and immunochromatographic sensors. The functional modification strategies and sensing mechanisms of these carbon-based nanomaterials for detecting typical agricultural and food contaminants, including heavy metal ions, pesticide residues, mycotoxins, antibiotics and food additives, are elaborated in detail. This work comprehensively integrates the intrinsic characteristics of materials, fundamental sensing principles, typical application cases and existing technical limitations in this field, and establishes a complete research framework. It aims to provide a theoretical basis and technical references for developing novel high-sensitivity, rapid and portable detection technologies for agriculture and food safety, and to promote the large-scale application and innovative development of carbon-based nanomaterials in precise food safety monitoring.

## 2. Major Carbon-Based Nanomaterials and Their Properties

### 2.1. Graphene and Its Derivatives

**Figure 3:**
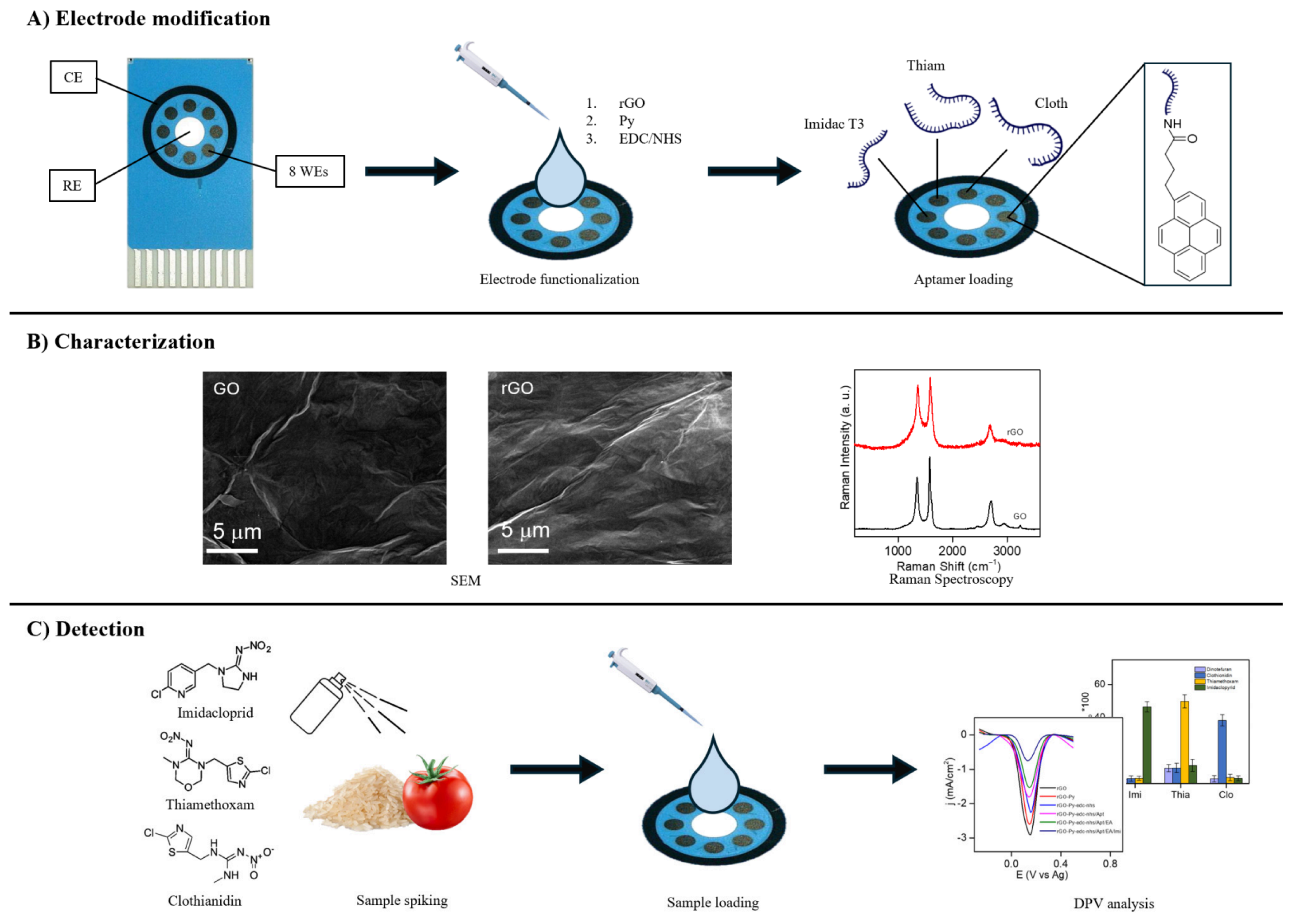
Reduced GO electrochemical aptasensor for multiplexed detection of neonicotinoids in food [45].

Graphene is a two-dimensional monolayer carbon material that features exceptional electrical conductivity, ultra-large specific surface area, and superior mechanical properties. As a critical derivative of graphene, graphene oxide (GO) is abundant in surface-active functional groups including hydroxyl and –COOH. Such structural characteristics enable the grafting of various functional units {e.g., L-cysteine, AuNPs, and molecularly imprinted polymers (MIPs)} through covalent and noncovalent modification strategies, which effectively enhances the specific recognition capability of materials toward target analytes [40]. Graphene-based materials have versatile applications in analytical detection. They can be engineered into ECS, where electrode modification with graphene-based composites allows the simultaneous detection of heavy metal ions such as Cd^2+^ and Pb^2+^ [41]. Moreover, fluorescent sensing probes based on the FRET effect of GO can be fabricated for the precise detection of zearalenone (ZEN) [42]. In addition, flexible wearable sensors constructed from graphene-based materials are capable of real-time monitoring of plant transpiration, supporting colorimetric and immunosensing analysis [43]. Apart from sensing applications, graphene-based materials also exhibit excellent performance in the adsorption and removal of organic contaminants and heavy metal ions from aqueous and environmental media [44]. Owing to their outstanding physicochemical properties and sensing performances, graphene materials have been extensively applied in multiple research areas, including electrochemical biosensors for heavy metal and pesticide detection (Figure 3) [45] and optical probes for pathogen identification, as well as being applied in the adsorption and detection of environmental pollutants [46,47]. These materials hold great promise for the development and practical deployment of high-sensitivity, fast-response, and miniaturized detection technologies.

### 2.2. Carbon Nanotubes

**Figure 4:**
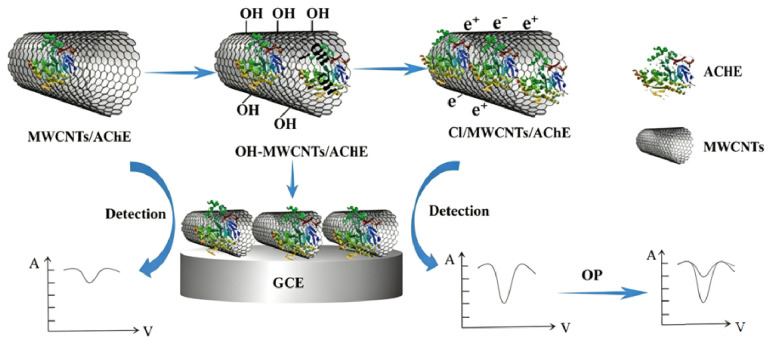
Nanomaterial-based SERS platform: substrates, enhancement mechanisms, and applications [24].

CNTs and their derivatives, with MWCNTs as the most representative form, possess a one-dimensional tubular structure, high aspect ratio, superior electron transport capacity, and distinctive porous hollow architecture. These unique structural and functional properties significantly facilitate interfacial electron transfer, thereby boosting the sensing sensitivity of corresponding sensors. Impressively, the LOD of MWCNT-based sensing platforms for kanamycin in milk can reach as low as 1.12 × 10^−8^ mg/g (Figure 4) [24]. To further expand their functionality, CNTs can be modified through covalent and noncovalent strategies to integrate diverse functional moieties and active units, including β-cyclodextrin, DES, AuNPs, ionic liquids, and -NH_2_. Such functionalization effectively strengthens the specific adsorption and molecular recognition of target analytes, substantially improving the selectivity of sensing assays [48]. Functionalized CNT-based materials exhibit versatile applicability in agricultural and food safety analysis. In electrochemical sensing, UiO-66-NH_2_@MWCNT composites enable the precise detection of Cd^2+^ in meat samples [49], while microelectrode arrays modified with β-cyclodextrin-functionalized MWCNTs (β-CD/MWCNTs) achieve the efficient quantification of imidacloprid residues in vegetables [50]. For colorimetric immunoassays, Au-decorated MWCNTs (Au/MWCNTs) can serve as nanozyme probes for the rapid detection of kanamycin in milk [35]. In terms of sample pretreatment, DES@MWCNTs act as high-efficiency QuEChERS sorbents for the purification of multiple pesticide residues in wolfberry samples [12]. Moreover, magnetically functionalized MWCNTs (Fe_3_O_4_-MWCNTs) coupled with UPLC-MS/MS allow the simultaneous screening and detection of various mycotoxins, delivering high sensitivity, excellent anti-interference capability, and favorable practicability for real-sample analysis [14,51]. Benefiting from their exceptional structural advantages and comprehensive performances, CNTs have been widely adopted in multiple scenarios, including electrochemical biosensors for rapid pesticide residue detection, gas sensing for agricultural product storage monitoring [52], and environmental heavy metal adsorption and remediation.

### 2.3. Carbon Quantum Dots

CQDs and their derivatives, represented by GQDs and nitrogen-doped CQDs (N-CQDs), are zero-dimensional carbon nanomaterials with particle diameters of less than 10 nm. They integrate excellent fluorescent performance, low biotoxicity, superior photostability, and remarkable pH responsiveness, thus possessing unique application advantages in the field of sensing detection [53]. The sensing performance of CQDs can be substantially optimized through various functional modification strategies. On the one hand, heteroatom doping with nitrogen and sulfur can effectively improve their fluorescence quantum yield and optimize optical sensing capabilities [54,55]. On the other hand, CQDs modified with vinyl phosphoric acid can be coupled with magnetic MIPs with homologous functional groups to construct specific fluorescent sensing systems for the detection of organophosphorus pesticides represented by triazophos [56]. Furthermore, the hybridization of CQDs with functional materials such as AuNPs, copper nanoclusters (Cu NCs), magnetic nanoparticles (MNPs), and nucleic acid aptamers enables the fabrication of ratiometric fluorescent probes and multimodal sensing platforms, which further improves the detection accuracy and anti-interference performance of sensing systems [57,58]. CQD-based functional materials exhibit versatile applicability and prominent practicability in agricultural and food safety analysis. Cu NCs@N-CQD ratiometric fluorescent probes realize the high-sensitivity detection of Pb^2+^ in laver samples [59], while CQD-MNP composite probes achieve the rapid recognition and detection of Escherichia coli in milk [60]. Dual-mode colorimetric/fluorescent sensors fabricated from CQDs and AuNPs are capable of the precise determination of malathion residues in cabbage [58]. In addition, nitrogen-doped graphene quantum dot composites (NGQDs-NH_2_-Ru@SiO_2_) can be constructed as electrochemiluminescence (ECL) sensing platforms for the efficient detection of ZEN [61]. Acid-responsive N-CQDs also support the rapid monitoring of milk freshness via visual fluorescent response [62]. All these sensing platforms feature high sensitivity, low cost, and excellent adaptability to real-sample matrices. As one of the core functional materials in fluorescent sensing, CQDs have been extensively utilized in the visual detection of pesticide residues, high-sensitivity identification of heavy metal ions, and bioimaging analysis. They provide efficient and intuitive technical support for rapid food safety screening and environmental pollutant monitoring, showing great application potential in high-efficiency and accurate analytical detection [63,64].

### 2.4. Biomass-Derived Carbon

Biomass carbon (BC) materials and their derivatives, such as BC aerogels and biochar, possess the prominent advantages of abundant sources, green renewability, and low cost, together with high specific surface area, well-developed pore structures, and rich surface-active functional groups. A variety of agricultural wastes, including sisal, bagasse, chestnut shells, and bamboo fibers, can be adopted as sustainable precursors for the fabrication of BC materials [65,66]. The functional performance of such materials can be effectively optimized via multiple modification strategies, including high-temperature carbonization activation, in situ growth modification, chemical grafting, and magnetic composite modification. Specifically, layered double hydroxide (LDH) nanosheets can be grown in situ on the material surface, while active functional groups such as sulfonic acid groups, –COOH, and thiol groups can be introduced through chemical modification. Additionally, composite modification with MNPs (e.g., Fe_3_O_4_) can be performed to significantly enhance the specific adsorption and recognition capability toward targeted contaminants [67,68]. Benefiting from their comprehensive and superior performance, BC materials have diversified application prospects in environmental remediation and analytical detection fields [69]. In terms of pollutant adsorption and removal, LDH-functionalized BC fibers achieve the efficient adsorption of fluoride in aqueous solutions with a high adsorption capacity of 15.21 mg/g [70]. Sodium lignosulfonate/carboxymethyl cellulose/BC composites (SLS/CMC/BC) enable the simultaneous removal of Pb^2+^ and methylene blue organic dyes from water environments [71]. Superhydrophobic BC aerogels (SHPC-200) exhibit excellent cycling stability, maintaining efficient oil–water separation performance even after 10 consecutive cycles [72]. In the field of catalysis, lignin-assisted chestnut shell carbon-supported molybdenum disulfide (MoS_2_) composites deliver outstanding catalytic performance for hydrogen evolution reactions (HERs) with a low overpotential of 86.84 mV [73]. Featuring environmental friendliness, low cost, and excellent regenerability, BC materials have been extensively applied in multiple research scenarios, including the adsorption and elimination of environmental pollutants, electrochemical biosensors for the detection of heavy metals and pesticide residues, and the rapid enrichment and detection of harmful substances in food matrices. They provide crucial technical support for the development of green and sustainable food and environmental detection technologies.

### 2.5. Other Emerging Carbon-Based Materials

In addition to the above conventional carbon-based materials, emerging two-dimensional carbon-based functional materials represented by g-C_3_N_4_ and MXenes (typically Ti_3_C_2_Tx) have become research hotspots in agricultural and food safety detection due to their unique physicochemical properties. As a typical two-dimensional layered semiconductor material, g-C_3_N_4_ exhibits excellent chemical stability, a distinctive electronic band structure, and low biotoxicity, conferring great potential for photoelectric sensing applications [74]. The photoelectric performance of g-C_3_N_4_ can be effectively optimized through functional modification strategies, including non-metallic doping with nitrogen and sulfur, as well as composite hybridization with quantum dots and metal oxides, which substantially improve its sensing and detection capabilities [75]. Currently, g-C_3_N_4_-based composites have been widely deployed in diverse sensing systems. A photoelectrochemical sensing platform based on g-C_3_N_4_ enables the efficient detection of aflatoxin B1 [76]. g-CNQDs@Zn-MOF composite fluorescent sensing systems achieve the precise and simultaneous detection of two typical pesticide residues, namely nitenpyram (NTP) and imidacloprid (IMI), in aqueous media [77]. Furthermore, g-C_3_N_4_-based fluorescent probes support the sensitive recognition and detection of hydrogen peroxide (H_2_O_2_) [78]. As an emerging class of two-dimensional transition metal carbides/nitrides, MXenes integrate metallic-level electrical conductivity, favorable hydrophilicity, ultra-large specific surface area, and abundant surface terminal functional groups. They also possess remarkable characteristics such as high specific capacitance, tunable electromagnetic properties, and superior surface hydrophilicity, demonstrating prominent advantages for sensing and electrochemical applications [79]. MXenes can be functionally modified via multiple approaches, including in situ polymerization, silanization covalent grafting, and aptamer physical adsorption, to further strengthen their specific recognition and sensing performance. Modified MXene materials deliver an ultralow LOD and excellent selectivity in various sensing and detection technologies. For instance, electrochemical sensing platforms constructed from GR5 DNAzyme/Ti_3_C_2_Tx are applicable for Pb^2+^ detection; AgNR/MXene composites can be fabricated into surface-enhanced Raman scattering (SERS) platforms for the efficient determination of polychlorinated biphenyls; and CQDs-apt-cDNA/MXene FRET probes enable the accurate identification of dimethoate pesticide residues [11]. Overall, emerging carbon-based materials including g-C_3_N_4_ and MXenes feature designable structures, tunable performances, and superior sensing capabilities. They open up innovative avenues for the development of high-sensitivity, multi-dimensional, and multifunctional detection technologies for agricultural and food contaminants.

## 3. Applications of Carbon Materials in Food Safety Detection

### 3.1. Detection of Heavy Metal Ions

Carbon-based nanomaterials possess superior physicochemical properties and have been widely applied in the trace detection of heavy metal ions in food. Electrochemical and fluorescence sensing stand out as the two most mainstream and efficient techniques, enabling highly sensitive, rapid and accurate analysis of heavy metal contaminants.

In the field of electrochemical sensing, Li et al. constructed a composite electrochemical sensing platform based on UiO-66-NH_2_@MWCNTs. The large specific surface area and abundant amino active sites of the metal–organic framework UiO-66-NH_2_, together with the outstanding electron transport capacity of CNTs, greatly improved the sensing response efficiency of the electrode interface, realizing the highly sensitive detection of Cd^2+^ in meat samples. After parameter optimization, the linear detection range for Cd^2+^ was 5 × 10^−7^~1.7 × 10^−4^ mg/g with an LOD as low as 2 × 10^−7^ mg/g. Moreover, the sensor exhibited strong anti-interference capability and excellent stability, making it suitable for rapid and accurate detection of trace cadmium in food [49].

For fluorescence sensing, Shi et al. developed a ratiometric fluorescent probe of Cu NCs@N-CQDs. Benefiting from the dual-signal self-calibration mechanism and aggregation-induced emission enhancement effect, this probe effectively eliminated environmental interferences inherent in single-signal detection and realized ultrasensitive determination of Pb^2+^ in laver samples. The linear range for Pb^2+^ was 10^−5^–0.0025 mg/g and the LOD reached 3.1 × 10^−6^ mg/g, with a relative standard deviation lower than 5%. Featuring satisfactory selectivity and repeatability, this work provides an innovative and efficient fluorescence sensing approach for the trace screening of Pb^2+^ in food [59].

In addition, emerging carbon-based materials such as MXenes also show distinctive advantages in heavy metal detection. A modified electrode based on GR5 DNAzyme/Ti_3_C_2_T_x_ composites was developed for the specific detection of Pb^2+^ in chicken samples. In the detection process, Pb^2+^ triggers the specific cleavage of substrate DNA by DNAzyme, which further enhances the adsorption capacity and electrochemical response of MXenes and increases the oxidation peak current. The corresponding linear range was 1.036 × 10^−7^~6.6304 × 10^−6^ mg/g. Integrating the high conductivity of MXenes and the specific biorecognition of DNAzyme, this sensor achieves highly sensitive and selective detection of Pb^2+^, and provides a new technical support for on-site rapid screening of heavy metals in food [11].

### 3.2. Detection of Pesticide Residues

Owing to their excellent physicochemical and sensing properties, carbon-based nanomaterials have been widely adopted for the detection of pesticide residues in food. Combined with electrochemical sensing, fluorescence sensing, colorimetric analysis and sample pretreatment techniques, these materials enable highly sensitive, rapid and accurate determination of various pesticide residues [80,81].

Zhu et al. fabricated modified electrodes based on defective graphene nanoribbons (DGNRs). Taking advantage of the large specific surface area, high electrical conductivity and abundant defective active sites of DGNRs, a high-performance electrochemical sensing system was constructed for the ultrasensitive detection of methyl parathion. The sensor exhibited a linear range of 2.6321 × 10^−9^~6.58025 × 10^−6^ mg/g with an LOD of 1.1318 × 10^−9^ mg/g. When applied to water and fruit samples, the spike recovery ranged from 95.7% to 106.4%. This work provides a novel low-cost and efficient sensing strategy for the detection of organophosphorus pesticide residues [82].

In the field of visual dual-mode detection, Shi et al. established a dual-signal sensing platform of CQDs-AuNPs integrating fluorescence and colorimetry. The combination of two detection signals effectively improved the anti-interference ability and practicality of the system, realizing visual and accurate detection of malathion in Chinese cabbage. The linear range covered 3.3036 × 10^−10^~3.304 × 10^−3^ mg/g. The LODs were 4.29468 × 10^−11^ mg/g for the fluorescence mode and 1.94912 × 10^−10^ mg/g for the colorimetric mode, and the spike recoveries were 88.7–107.6%. This platform integrates high sensitivity, visualization and convenience, and is well suited for on-site rapid detection [58].

Novel carbon-based composites also show excellent performance in photoelectrochemical sensing. Jiang et al. developed a self-powered photoelectrochemical sensor based on a Ti_3_C_2_/AgBr Schottky structure. Relying on the metal–ligand charge transfer effect at the material interface, the sensor achieved highly sensitive detection of chlorpyrifos. Without the use of precious metals and biorecognition elements, the detection cost was greatly reduced. Its linear range was 1.0 × 10^−12^~1.0 × 10^−9^ mg/g and the LOD was as low as 3.3 × 10^−13^ mg/g. The sensor can be effectively applied to the quantitative determination of chlorpyrifos residues in fruits, vegetables and water samples [83].

**Figure 5:**
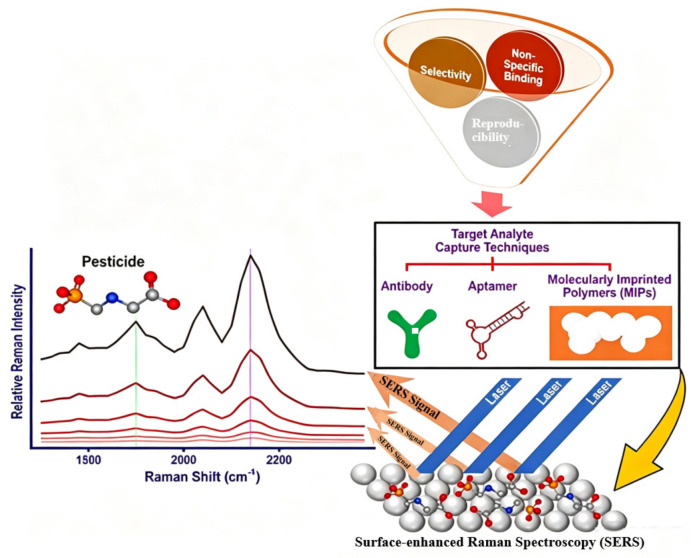
Challenges in SERS-based pesticide detection and plausible solutions [20].

Carbon-based nanomaterials also serve as ideal substrates for surface-enhanced Raman spectroscopy (SERS) sensors. When composited with gold and silver noble metals, they synergistically amplify plasmonic signals to enable ultrasensitive screening of pesticide residues. The limit of detection for chlorpyrifos in apples reaches 1.0 × 10^−5^ mg/g, while that for thiram in tea is as low as 2.4 × 10^−11^ mg/g. After functional modification, these materials can suppress non-specific adsorption from sample matrices and support the simultaneous detection of multiple pesticide components (Figure 5) [20].

### 3.3. Detection of Mycotoxins

With excellent structural and sensing properties, carbon-based nanomaterials have been widely utilized for the trace detection of mycotoxins in food. Combined with electrochemical sensing, fluorescence sensing, colorimetric analysis and sample pretreatment techniques, these materials enable the highly sensitive, rapid, quantitative screening of various mycotoxins.

Li et al. fabricated an aptamer-based fluorescent sensing system based on FRET using upconversion nanoparticles (UCNPs) and functionalized GO (FGO) for the specific detection of ZEN. In this system, UCNPs served as stable fluorescence donors and FGO acted as an efficient fluorescence quencher. The specific recognition of targets by aptamers triggered the recovery of fluorescence signals, thus realizing the accurate quantification of ZEN. The sensor had a linear range of 5 × 10^−9^~1 × 10^−4^ mg/g with an LOD of 1.8 × 10^−9^ mg/g. It also possessed high specificity and favorable stability, which is suitable for the rapid screening of ZEN in grain samples [42]. Li et al. constructed a self-enhanced ECL nanosystem based on nitrogen-doped GQDs (NGQDs) and aminated Ru-doped silica. Benefiting from the intrinsic self-enhanced ECL effect, this system required no additional coreactants. Meanwhile, it shortened the electron transfer path and improved signal response efficiency, achieving the ultrasensitive detection of ZEN. The linear range was 1.0 × 10^−14^~1.0 × 10^−8^ mg/g and the LOD reached 1.0 × 10^−15^ mg/g, with the sensitivity improved by three orders of magnitude compared with conventional methods. This platform can be stably applied to the trace analysis of ZEN in practical food samples such as corn flour [61].

Emerging two-dimensional carbon materials represented by MXenes also show prominent advantages in mycotoxin detection. MXene-based aptasensors make full use of the material’s high conductivity, large specific surface area and easy functionalization, and realize the highly sensitive detection of typical mycotoxins including aflatoxin and ZEN. As an ideal sensing substrate and signal amplification unit, MXene greatly improves detection sensitivity, with LODs down to femtogram or picogram levels. Such sensors possess outstanding specificity and anti-interference ability, and are applicable to trace mycotoxin detection in complex food matrices such as grains [11].

Aiming at the ultrasensitive detection of ochratoxin A (OTA), Li et al. developed a high-performance fluorescent aptasensing platform with a cascade signal amplification strategy. Polyvinylpyrrolidone-modified GO (GO@PVP) was adopted to adsorb free fluorescent aptamers and reduce background fluorescence interference. Silver nanoparticle-embedded gel (AgNPs@gel) worked as a metal-enhanced fluorescence substrate for primary signal amplification. Combined with the cyclic cleavage reaction of DNase I, a dual-signal amplification mechanism was established to further boost the detection sensitivity. This method had a linear range of 3.33 × 10^−9^~5.56 × 10^−8^ mg/g and an LOD as low as 3.33 × 10^−9^ mg/g. With high specificity and good stability, it can be used for trace OTA detection in food grains, alcoholic beverages and other real samples, providing a novel and efficient nanosensing strategy for ultrasensitive analysis of food mycotoxins [84].

### 3.4. Detection of Antibiotic and Drug Residues

In the detection of antibiotic and veterinary drug residues in food, carbon-based nanomaterials can be combined with various analytical techniques including colorimetric immunoassay, electrochemical sensing and immunochromatography to achieve highly sensitive, rapid screening and accurate quantification of diverse drug residues, showing great application potential in food drug risk control [85].

Meng et al. synthesized Au/MWCNT nanohybrids. Owing to their excellent peroxidase-like catalytic activity, the composites can replace natural enzymes with poor stability and high cost to establish a direct competitive immunoassay system for the ultrasensitive detection of kanamycin residues in milk. With favorable catalytic performance and structural stability, this method presented an LOD of 11.2 × 10^−9^ mg/g and a linear range of 17.2 × 10^−9^–64.4 × 10^−9^ mg/g. The spike recoveries ranged from 94.3% to 124.5%, which met the requirements for the screening of trace kanamycin in complex food matrices [35].

Using multi-walled CNTs (MWCNTs) as solid-phase adsorption carriers, Du et al. developed two immunoassays for the rapid detection of gentamicin residues in milk, namely quantitative centrifugal detection and qualitative filtration detection. The LOD of the quantitative method was 4.8 × 10^−8^ mg/g, and the visual detection limit of the qualitative method was 1.0 × 10^−7^ mg/g, both far below the maximum residue limit of 1.0 × 10^−4^ mg/g stipulated by the European Union. The two methods were verified with five batches of commercial milk samples and exhibited good stability and practicality. They provide a simple, sensitive and high-throughput technical platform for the detection of trace antibiotic residues in food [26].

For on-site rapid detection, Wang et al. prepared specific nanoprobes based on AuNPs and fabricated colloidal gold immunochromatographic test strips with monoclonal antibodies for the rapid detection of lomefloxacin residues in meat products. In this test strip, specific antibodies were labeled with colloidal AuNPs, and the targeted detection was realized via specific antigen–antibody immune recognition. The cross-reactivity towards structural analogs was lower than 0.1%, indicating excellent detection specificity. Meanwhile, the half-maximal inhibitory concentration (IC_50_) of the corresponding indirect competitive ELISA (ic-ELISA) was 9.3 × 10^−7^ mg/g with a linear range of 3.8 × 10^−7^~2.3 × 10^−6^ mg/g. The visual detection limit of the test strip was 2.5 × 10^−6^ mg/g, and the whole detection process could be completed within 10 min, which fully satisfied the demand for on-site rapid screening of food samples [86].

Apart from immunoassays, gold nanomaterials can also serve as fluorescent probes to construct enzyme-based rapid detection platforms for food residues. Wang et al. adopted glutathione-modified gold nanoclusters (GSH-AuNCs) with outstanding fluorescence performance and high photostability as fluorescent nanoprobes. Combined with genetically engineered recombinant carboxylesterase, a novel detection system was established based on the inner filter effect and enzyme inhibition principle. Organophosphorus pesticides can inhibit enzyme activity and further change the fluorescence signal of the system. This method possesses high sensitivity and strong anti-interference capability, and delivers reliable results in actual apple sample detection, offering a new strategy for rapid pesticide residue detection [87].

### 3.5. Detection of Food Additives and Illegal Additives

In the field of detecting food additives and illegal adulterants, carbon-based nanomaterials combined with electrochemical sensing, fluorescence sensing, colorimetric analysis and other techniques enable highly sensitive and rapid trace screening of legal additives and prohibited substances, providing strong technical support for food quality and safety supervision.

Guo et al. developed a composite electrochemical sensor based on GO/AgNPs-MIPs for the specific detection of sunset yellow, a synthetic colorant, in soft drinks. MIPs offer abundant specific recognition sites to endow the sensor with excellent targeting capability, while silver nanoparticles improve electrode conductivity and amplify electrochemical signals. The synergistic effect of these components greatly enhances the detection performance. The sensor exhibited two well-defined linear ranges of 0.04524~0.27144 mg/g and 0.27144~5.4288 mg/g, with an LOD of 0.009048 mg/g. Satisfactory recoveries were obtained in real beverage samples. This work presents a novel strategy for the highly selective and sensitive detection of synthetic colorants in food [40].

To meet the demand for on-site visual detection of illegal additives, Zou et al. established a fluorescent sensing platform using Au@CQD nanocomposites. Melamine can induce the aggregation of AuNPs and enhance the fluorescence of the system. Combined with smartphone imaging and portable spectral detection, this method realized visual and quantitative analysis of melamine residues in milk. A good linear relationship was observed within 0.12612~1.2612 mg/g, with an LOD of 0.000454 mg/g and a limit of quantification (LOQ) of 0.001513 mg/g. The spike recoveries for milk samples were 102.75–105.64%, and the results were highly consistent with those obtained by HPLC. This method integrates ultrahigh sensitivity and convenient on-site visual detection [88]. Zhang et al. synthesized low-toxicity and water-soluble CQDs via a one-pot hydrothermal method. Using CQDs to label specific antibodies, a fluorescence-linked immunosorbent assay was established for the trace detection of morphine, a characteristic marker of illegal poppy shell addition in hot pot bases. Compared with traditional CdSe/ZnS quantum dots, the as-prepared CQDs feature low toxicity, superior biocompatibility and environmental friendliness. Under optimal conditions, the linear range was 3.2 × 10^−7^~0.010 mg/g and the LOD was 3 × 10^−7^ mg/g, with spike recoveries of 85–97%. It provides a new green, sensitive and rapid tool for screening illegal additives in food [89].

**Figure 6:**
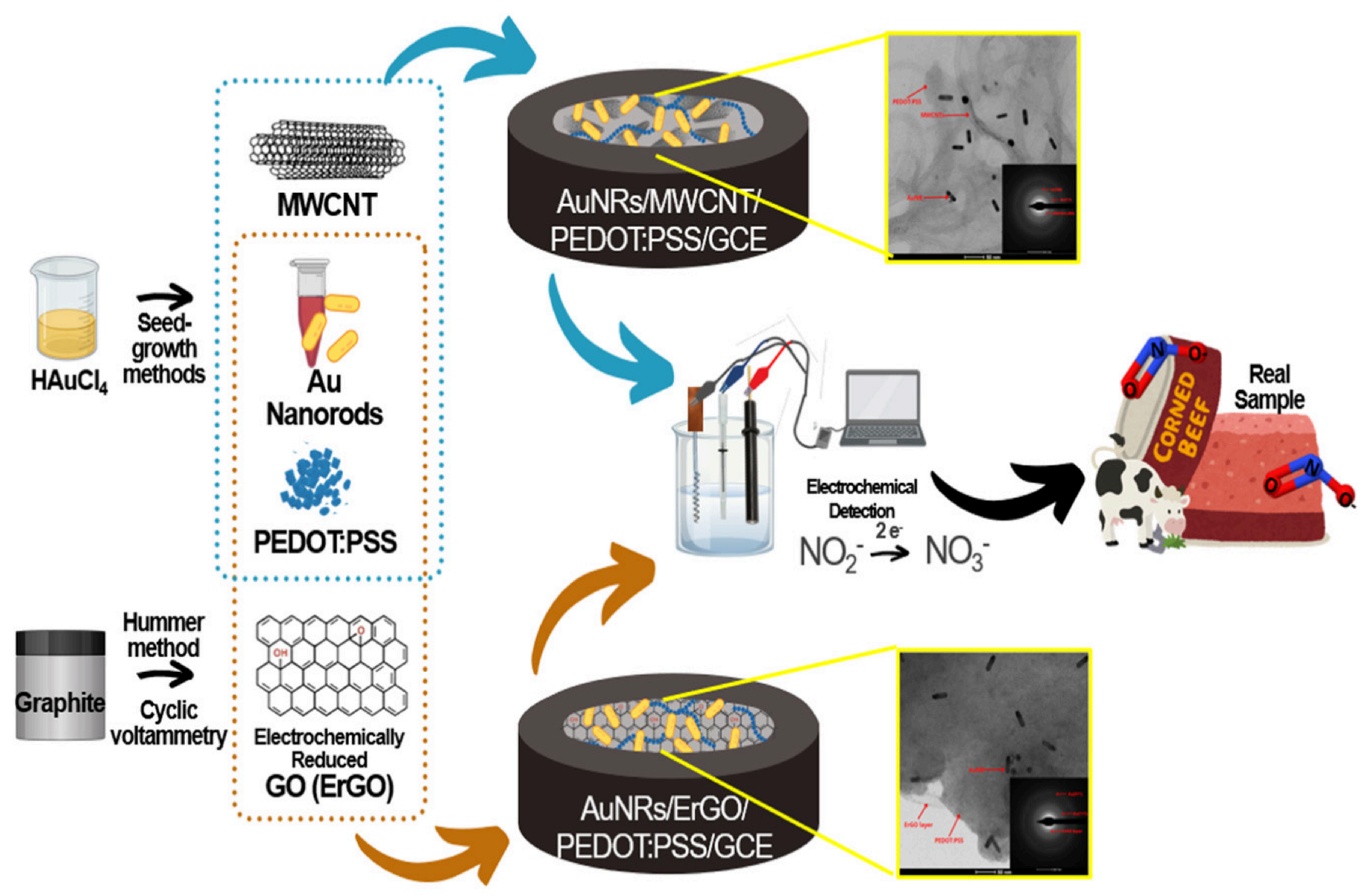
Two gold nanorods/carbon nanomaterials/PEDOT: PSS-based ECS for nitrite detection in processed meat [90].

For the rapid detection of nitrite in food, Putra et al. fabricated two types of gold nanorod-modified glassy carbon electrodes. The synergistic effect of different components enhanced the electrocatalytic activity, and the electrodes were applied to nitrite detection in meat products by differential pulse voltammetry. Comparative experiments demonstrated that the electrode modified with gold nanorods, RGO and poly(3,4-ethylenedioxythiophene)-poly(styrenesulfonate) (AuNRs/MWCNT/PEDOT: PSS) possessed a larger electrochemically active area. It showed a linear range of 9.202 × 10^−3^~4.601 mg/g and an LOD of 3.681 mg/g, with favorable anti-interference ability and stability. The detection results were in good agreement with conventional methods (Figure 6) [90].

On the basis of carbon-based composite electrodes, Han et al. prepared a nanocomposite by combining multi-walled CNTs (MWCNTs) with AuNPs and polymelamine to construct an electrochemical sensor for nitrite detection. With their large specific surface area and excellent conductivity, CNTs can effectively load metal nanoparticles and accelerate electron transfer, thus further improving the catalytic performance. The sensor had a linear range of 0.018404~67.86475 mg/g and an LOD as low as 0.001886 mg/g. Optimized via layer-by-layer self-assembly technology, this novel method shows strong anti-interference performance and achieves good recovery in real-sample analysis [91].

### 3.6. Monitoring of Food Freshness and Quality

In the field of intelligent monitoring of food freshness and quality, carbon-based nanomaterials combined with cutting-edge technologies such as fluorescence sensing, colorimetric analysis and flexible wearable sensing enable real-time, non-destructive and visual detection of food quality. They provide innovative technical approaches for food preservation monitoring and quality evaluation in smart agriculture [92].

Shi et al. fabricated paper-based and starch gel film fluorescence sensing systems using acid-responsive N-CQDs, and established a visual detection method for milk freshness. The fluorescence intensity of N-CQDs showed a good linear correlation with milk acidity in the range of 11.6–34.2 °T, with a determination coefficient of R_2_ = 0.996. This method requires no complicated sample pretreatment. Under ultraviolet light, the deterioration degree of milk can be directly identified by observing fluorescence color changes with reference to standard color cards. Featuring simple operation and portable equipment, this approach is highly applicable for high-sensitivity on-site evaluation of food freshness [62].

Zhai et al. also adopted CQDs as core functional materials. CQDs were immobilized on hydrophobic polyvinylidene fluoride (PVDF) films via electrospinning to prepare CQDs@PVDF ratiometric fluorescent sensing films, which were applied to real-time spoilage monitoring of fresh meat and aquatic products including beef, pork and shrimp under high-humidity preservation at 4 °C. Volatile amines such as trimethylamine released during food spoilage can trigger protonation reaction with CQDs, leading to a reversible fluorescence color transition from yellow-green to blue for visual quality discrimination. The limits of detection for TMA were 0.061474 mg/g for CQDs@PVDF films and 0.124131 mg/g for CQD/PVDF films. In particular, CQDs@PVDF films possessed superior structural stability and response reversibility, serving as a reliable sensing platform for non-destructive and real-time quality monitoring in intelligent food packaging [93].

Graphene-based flexible wearable sensors present unique advantages in in situ and non-destructive detection for crop quality and physiological status monitoring. Mao et al. developed a flexible wearable sensing device based on polydimethylsiloxane–graphene oxide–sodium dodecyl sulfate composite substrates. GO acted as a humidity-sensitive material and a platinum thin film served as a temperature-sensitive component, which could in situ and non-destructively collect key physiological parameters of plant leaves including temperature, humidity and leaf vapor pressure deficit. The sensor exhibited ultrahigh sensitivity, with a humidity sensitivity of 4456 pF/%RH and a temperature sensitivity of 3.93 Ω/°C. It also had fast response, strong anti-interference capability and good adhesion to leaf surfaces, enabling accurate in situ monitoring of the physiological status of tomato leaves. This work offers a novel strategy for precise perception of crop physiological conditions and quality prediction in smart agriculture [43].

In addition, carbon-based nanofillers can optimize the microstructure of intelligent packaging films, enhancing their gas-sensing response and UV-blocking performance. These films are capable of rapidly detecting volatile amines and hydrogen sulfide generated during food spoilage, with the limit of detection for ammonia reaching the ppm level and the response time lasting only several minutes. This provides strong nanosensing support for non-destructive monitoring of fresh food [94].

## 4. Applications of Carbon Materials in Agriculture and Environmental Remediation

### 4.1. Adsorption of Heavy Metal Ions

Carbon-based nanoadsorbents possess excellent structural and adsorption properties and show prominent advantages in the remediation of heavy metal pollution in agricultural soil and water environments. Novel carbon materials such as functionalized BC, modified CNTs and magnetic composite aerogels can efficiently enrich and remove heavy metal ions from water, and have become a research hotspot in green environmental remediation [95].

Wang et al. synthesized a composite carbon adsorbent (SLS/CMC/BC) by using waste cotton as the carbon source, followed by grafting CMC and sodium lignosulfonate via radical polymerization. The as-prepared material can simultaneously remove heavy metal ions and organic dye contaminants. In single-pollutant systems, the maximum adsorption capacities for Pb^2+^ and methylene blue reached 204 mg/g and 113 mg/g, respectively. In the binary system containing Pb^2+^ and methylene blue, the coexistence of methylene blue further promoted the adsorption of Pb^2+^, with the adsorption capacity increasing to 237.3 mg/g, while Pb^2+^ exerted no obvious inhibitory effect on the adsorption of methylene blue. Mechanism analysis revealed that electrostatic adsorption and surface complexation were the dominant adsorption mechanisms. Moreover, the composite still maintained satisfactory adsorption performance after five adsorption–desorption cycles, indicating good cyclic stability [71].

Using agricultural waste bagasse as the fibrous raw material and chitosan as the matrix, Li et al. fabricated magnetic dual-substrate composite aerogel Fe_3_O_4_@bagasse@chitosan (FBC) via hydrothermal reaction and freeze-drying technology for efficient removal of hexavalent chromium (Cr(VI)) from water. Under the optimal conditions of 65 °C, pH = 3 and initial contaminant concentration of 0.25 mg/g, the maximum adsorption capacity toward Cr(VI) was 60 mg/g, which was much higher than that of conventional adsorbents (approximately 41 mg/g). The synergistic effect derived from the reduction of Fe_3_O_4_ as well as electrostatic adsorption by amino groups and –COOH on chitosan dominated the adsorption process. Benefiting from good magnetic responsiveness, the material could be rapidly separated and recovered under an external magnetic field. Being eco-friendly, low-cost and reusable, this composite is a promising adsorbent for the treatment of Cr(VI)-containing wastewater [44].

Hao et al. prepared lignosulfonate–lysine composite hydrogels, which exhibited outstanding adsorption performance toward Cu^2+^ and Co^2+^ in aqueous solutions. The maximum adsorption capacities were up to 383 mg/g for Cu^2+^ and 286 mg/g for Co^2+^. Kinetic and mechanism studies demonstrated that the adsorption was mainly governed by chemical adsorption, accompanied by electrostatic interaction. Competitive inhibition was observed between Cu^2+^ and Co^2+^ during the adsorption process. After ten regeneration cycles, the adsorption efficiencies for Cu^2+^ and Co^2+^ remained at 54.61% and 60.1%, respectively. The hydrogel possesses excellent reusability and long-term service potential, providing a new functional material for economical and efficient treatment of heavy metal wastewater [96].

### 4.2. Removal of Organic Pollutants

In the remediation of organic pollution in agricultural and water environments, carbon-based nanomaterials such as BC aerogels and functionalized CNTs exhibit unique advantages including tunable porous structures and facile functional modification. These materials can efficiently adsorb and separate various organic pollutants in water, including oils, organic solvents and organic dyes, serving as ideal functional materials for environmental organic pollution remediation.

Zhang et al. prepared superhydrophobic BC aerogels via high-temperature carbonization using agricultural waste corn bracts as raw materials. The material possesses a well-developed three-dimensional porous structure with a specific surface area of 675.85 m^2^/g and a water contact angle of 152°, showing excellent hydrophobic and lipophilic properties. Its adsorption capacities for oils and organic solvents such as engine oil and n-hexane range from 77,670 to 143,630 mg/g. Moreover, the BC aerogel exhibits outstanding cyclic stability, retaining more than 90% of its original adsorption capacity after ten adsorption–desorption cycles. With stable hydrophobicity and reusability, it provides a green and efficient adsorbent for oil–water separation and oily wastewater treatment [97].

Furthermore, Zhang et al. fabricated Mg-Al layered double hydroxide (Mg-Al-LDHs)-functionalized BC aerogels using sisal fiber precursors for the efficient removal of selenium from water. The composite material has a specific surface area of 165.26 m^2^/g and a maximum selenium adsorption capacity of 73.6 mg/g, reaching adsorption equilibrium within 4 h with high removal efficiency. After nine regeneration cycles, the material still maintains favorable adsorption performance. Benefiting from the unique “memory effect” of LDHs, the composite also has potential for the derivatized removal of organic dyes in water, demonstrating excellent functional expandability [65].

## 5. Analysis of Sensing and Remediation Mechanisms of Carbon Materials

### 5.1. Electrochemical Sensing Mechanisms

Carbon-based nanomaterials exert three synergistic effects in electrochemical sensing systems, namely electrochemical signal amplification, immobilization of biorecognition elements, and enzyme-like catalytic enhancement. These three mechanisms collectively determine the sensitivity and specificity of sensors. Representative research cases for the detection of heavy metals, pesticides and mycotoxins in food from Section 3 are analyzed one by one in the following text, and the regulatory effects of the above mechanisms are verified with practical experimental data.

#### 5.1.1. Electrochemical Signal Amplification Effect

Graphene, CNTs and other carbon-based nanomaterials integrate an ultra-large specific surface area and excellent intrinsic conductivity. They can effectively optimize the electrode interface structure, accelerate interfacial electron transfer, reduce charge transfer resistance, and significantly improve the sensitivity of sensing detection [98]. Electrochemical impedance spectroscopy tests show that the charge transfer resistance of a bare glassy carbon electrode is approximately 200 Ω, while graphene modification reduces the interfacial resistance to 25 Ω, which directly verifies that graphene can optimize electrode conductivity and weaken interfacial mass transfer resistance [99]. This signal amplification mechanism serves as the core foundation of the simultaneous heavy metal detection system described in Section 3.1. Sun et al. fabricated an L-cysteine/graphene composite glassy carbon electrode (L-cys/GR-CS/GCE). The high-speed electron transport capacity of graphene was utilized to amplify the redox signals of cadmium and lead ions, and the specific coordination interaction between L-cysteine and heavy metals further enabled the simultaneous determination of trace Cd^2+^ and Pb^2+^ in honey and rice. The limits of detection for Cd^2+^ and Pb^2+^ were as low as 4.5 × 10^−7^ and 1.2 × 10^−7^ mg/g, respectively, and the obtained detection results were in great agreement with those from inductively coupled plasma optical emission spectrometry (ICP-OES) [41].

#### 5.1.2. Efficient Immobilization Carrier for Biorecognition Elements

Abundant active sites including carboxyl, amino and hydroxyl groups can be introduced onto the surface of carbon-based materials via doping, oxidation and grafting modification, which enables stable immobilization of biorecognition elements such as enzymes, aptamers and antibodies. This strategy addresses the drawbacks of traditional biological probes including easy inactivation and shedding, and improves detection specificity at the molecular recognition level.

The aptamer sensing system presented in Section 3.2 also relies on this mechanism. Oxygen-containing functional groups on reduced graphene oxide (rGO) can firmly immobilize aptamers targeting neonicotinoid pesticides through hydrogen bonding and electrostatic interactions. As illustrated in Figure 3, the rGO-based electrochemical aptasensor enables simultaneous identification of multiple pesticides such as imidacloprid and thiamethoxam in tomatoes and cereals. The stable immobilization of aptamers endows the sensor with outstanding specificity without obvious cross-interference [45].

This mechanism is further validated by the electrochemiluminescence (ECL) system for mycotoxin detection in Section 3.3. Amino groups on aminated graphene quantum dots can covalently bind ZEN aptamers. The stable immobilization of aptamers guarantees steady ECL signal output, and ultimately achieves femtogram-level ultrasensitive quantification of ZEN in grain samples [61].

#### 5.1.3. Enzyme-like Catalytic Enhancement Effect

**Figure 7:**
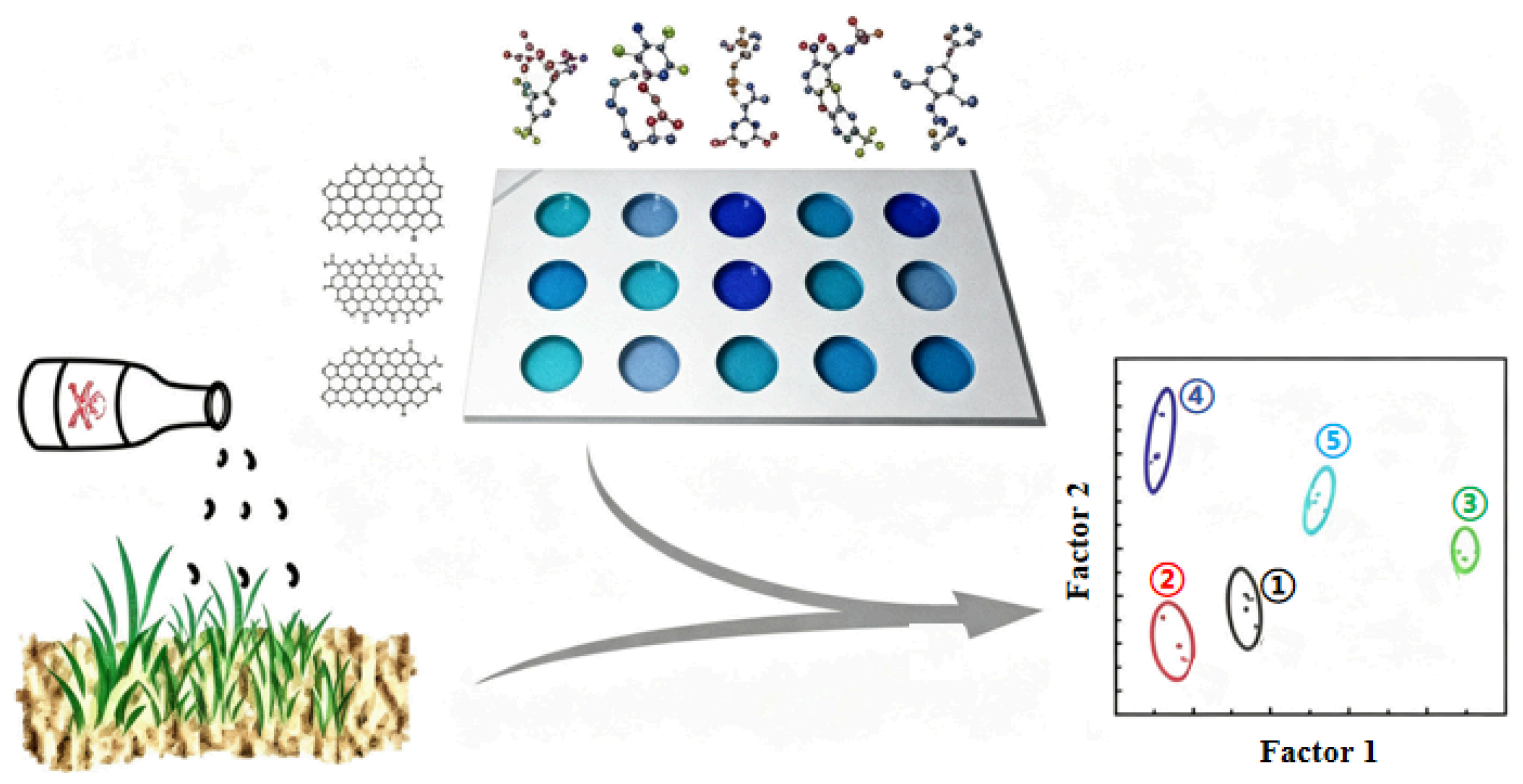
Nanozyme sensor arrays based on heteroatom-doped graphene for detecting pesticides [100].

Graphene, carbon nanotubes and noble metal–carbon hybrid materials possess intrinsic peroxidase-like catalytic activity, which can catalyze redox reactions of substrates and simultaneously amplify electrochemical voltammetric signals and colorimetric optical signals to doubly boost detection performance. Figure 7 visually demonstrates the distinct catalytic mechanisms between various pesticides and carbon-based nanozymes using a heteroatom-doped graphene nanozyme array. Carbon-based nanomaterials and their composites exhibit prominent nanozyme catalytic activity, which can efficiently catalyze substrate redox reactions, synchronously amplify electrochemical and colorimetric signals, and comprehensively improve the overall detection performance of sensors (Figure 7) [100]. As shown in Figure 7, heteroatom-doped graphene was fabricated into a nanozyme sensor array for pesticide detection. Distinct multicolor colorimetric responses of the array enable direct visualization of the differentiated catalytic interaction mechanisms between different pesticides and carbon-based nanozymes.

The kanamycin detection system for milk described in Section 3.4 serves as a typical application of this mechanism. Meng et al. synthesized Au/MWCNT nanohybrids. The excellent peroxidase-like activity of the composite was utilized to catalyze chromogenic substrates, replacing expensive and labile natural enzymes to construct a competitive immunosensing platform. The limit of detection was as low as 1.12 × 10^−8^ mg/g, satisfying the screening requirements for trace veterinary drug residues in milk [39].

Metalloporphyrin-functionalized multi-walled carbon nanotube composites (ZnTPP/MWCNTs) enhance the redox response of tert-butylhydroquinone via a multi-component synergistic catalytic mechanism. The reduction peak current reaches 277.0 μA, which is 50 times that of the bare electrode. The linear range covers 1.6622 × 10^−6^ to 0.16622 mg/g with a limit of detection of 4.48794 × 10^−6^ mg/g and spike recoveries ranging from 93.0% to 102.6%. Such a catalytic signal amplification effect is the core contributor to ultrasensitive detection [101].

The synergistic effect of the three components enables electrochemical sensors to achieve highly sensitive and selective detection.

### 5.2. Optical Sensing Mechanisms

Optical sensing based on carbon materials relies on three core mechanisms, namely fluorescence resonance energy transfer (FRET), photoinduced electron transfer (PET), and synergistic fluorescence–colorimetry dual-mode sensing. Carbon quantum dots, graphene oxide and carbon-based nanozymes act as fluorescence donors, fluorescence quenchers and catalytic chromogenic units, respectively. Combined with the detection cases of heavy metals, pesticide residues, mycotoxins and fresh food quality described in Section 3, this section elaborates on how the above mechanisms regulate fluorescence and colorimetric signals to realize visual quantification of pollutants one by one.

#### 5.2.1. FRET Mechanism

In FRET systems, carbon quantum dots function as fluorescence donors, while graphene oxide or gold nanoparticles serve as quenching acceptors. The specific binding between targets and aptamers alters the molecular distance within the sensing system, leading to reversible recovery of fluorescence signals and thus enabling quantitative detection [102].

The MXene-based fluorescent probe for dimethoate pesticides presented in Section 2.5 and Section 3.2 also operates on the FRET effect. In the carbon quantum dot–aptamer–complementary DNA/MXene system, MXene rapidly quenches the fluorescence of carbon quantum dots in the absence of target analytes. When dimethoate is present, the aptamer specifically binds to the target, disrupting the FRET interaction and restoring fluorescence intensity, which achieves specific identification of pesticide residues [11].

#### 5.2.2. PET Mechanism

Zero-dimensional carbon quantum dots act as electron carriers, and photoinduced electron transfer (PET) occurs under light irradiation to directly regulate the fluorescence intensity of the system. Electrochemiluminescence (ECL) systems constructed on this basis can further shorten the electron transfer pathway and greatly improve detection sensitivity [61,103].

The nitrogen-doped graphene quantum dot ECL sensing platform in Section 3.3 is a typical representative of this mechanism. The NGQDs-NH_2_-Ru@SiO_2_ system relies on the intrinsic self-enhanced ECL-PET effect and requires no additional coreactants. Its greatly shortened electron transfer pathway enables an ultralow limit of detection of 1.0 × 10^−15^~1.0 × 10^−9^ mg/g for ZEN, with three-orders-of-magnitude-higher sensitivity than conventional fluorescence methods. This platform can be stably applied to the detection of real corn flour samples [61].

#### 5.2.3. Dual-Mode Fluorescence–Colorimetric Synergistic Sensing Mechanism

Carbon-based nanozymes can simultaneously catalyze substrate chromogenic reactions. Combined with dual-channel cross-verification of fluorescence signals, they effectively mitigate optical interferences caused by pigments and suspended matter in food matrices and realize naked-eye visual on-site detection [102].

The dual-signal sensor for malathion in Chinese cabbage described in Section 3.2 fully verifies this synergistic mechanism. Shi et al. constructed a carbon quantum dot–gold nanoparticle fluorescence–colorimetric dual-mode platform. The presence of malathion simultaneously triggers changes in fluorescence intensity and solution color, and mutual calibration of the two signals drastically reduces matrix interference from fruits and vegetables. The limits of detection reach 4.29468 × 10^−11^ mg/g for the fluorescence mode and 1.94912 × 10^−10^ mg/g for the colorimetric mode, enabling visual quantitative on-site detection without large-scale instruments [58].

#### 5.2.4. Inner Filter Effect for Auxiliary Regulation of Optical Signals

The inner filter effect can assist in regulating optical signals and further improve the quantitative accuracy of fluorescent probes.

The enzyme-based fluorescent detection of organophosphorus pesticides in Section 3.4 relies on the inner filter effect. Glutathione-modified gold nanoclusters (GSH-AuNCs) serve as fluorescent probes. After organophosphates inhibit the activity of carboxylesterase, the inner filter effect of the system is weakened and the fluorescence intensity increases, thereby enabling rapid detection of pesticide residues in apple samples [87].

Compared with the fluorescence, SERS and colorimetric optical sensing systems described below, electrochemical sensors realize direct electron conduction at the interface and are free from matrix-induced optical interferences such as fluorescence quenching and light scattering. However, a single electrochemical signal lacks intuitive evidence for visual qualitative analysis. In contrast, fluorescence and colorimetric sensing allow on-site visual detection with the naked eye, yet they are susceptible to interference from colored substances in samples such as food pigments and humus. The four types of sensing systems differ greatly in their core working pathways, anti-interference capabilities and limit of detection ranges. The mechanistic differences among these detection systems are summarized in Table 1.

As can be seen from Table 1, the four carbon-based sensing systems exhibit fundamental differences in their core working mechanisms. Electrochemical sensors generate signals through electron conduction, while optical sensors regulate fluorescence and color development via photon energy transfer, and SERS relies on plasmon resonance to amplify characteristic spectra. A single contaminant can be detected by multi-mode hybrid sensing systems, such as the combination of carbon quantum dot-based fluorescence and carbon nanotube-based electrochemistry, which compensates for the shortcomings of individual sensing strategies. For instance, in the detection of pesticide residues in fruits and vegetables, electrochemical sensing enables accurate quantitative analysis, and fluorescent test strips achieve rapid on-site preliminary screening. The synergistic effect of these two mechanisms can greatly improve the detection accuracy in complex fruit and vegetable matrices.

### 5.3. Adsorption and Separation Mechanisms

Carbon-based adsorbents remove pollutants in farmland soil and water via three mechanisms: physical adsorption, chemical adsorption, and the synergistic combination of the two. These mechanisms also serve as the core theoretical basis for the QuEChERS sample pretreatment described in Section 3 and the remediation of heavy metal and organic contaminants in soil and water discussed in Section 4. Combining the remediation cases of biomass carbon, carbon nanotubes and magnetic aerogels presented in Section 4, this section elaborates on how material structures achieve efficient removal of pollutants through different adsorption mechanisms.

Physical adsorption: Hierarchical microporous and mesoporous structures capture oils and small molecular organic compounds through van der Waals forces, and superhydrophobic biomass carbon aerogels can store large amounts of organic liquids via their porous networks [104]. Meanwhile, the physical adsorption performance of biochar can be directionally optimized by adjusting pyrolysis temperature and raw material types, presenting high structural designability [105].

Chemical adsorption: Surface functional groups such as -COOH, -OH and -NH_2_ selectively bind heavy metal ions through complexation, electrostatic interaction and ion exchange. The adsorption capacity of modified biochar can be increased by more than four times [106]. Composite materials possess favorable magnetic separation performance and excellent cycling stability [107].

Physicochemical synergistic adsorption: The porous structure enriches pollutants physically [108], while surface functional groups realize harmless removal of pollutants via complexation and redox reactions [109,110]. Magnetic aerogels can first adsorb Cr(VI) and then reduce it to less-toxic Cr(III). Cyclodextrin-modified carbon nanotubes achieve host–guest inclusion and surface complexation simultaneously [44]. Carbon-based adsorbents with different structures remove pollutants in water and soil through distinct dominant adsorption mechanisms. Biomass-derived carbon mainly relies on physical porous adsorption, accompanied by complexation via surface functional groups. Carbon nanotubes enhance the encapsulation of organic pollutants through the confinement effect of hollow channels. Magnetic composite carbon aerogels achieve simultaneous reduction of heavy metals and magnetic separation. In addition, g-C_3_N_4_ and MXene can exert synergistic photocatalytic degradation effects. These materials differ markedly in adsorption mechanisms, adsorption capacities and cycling performance, and a comprehensive comparison is presented in Table 2.

As indicated by the mechanistic comparison in Table 2, a single type of carbon-based material can hardly efficiently remediate combined pollution of heavy metals and organic contaminants in farmland. The composite system consisting of biomass carbon aerogel and MXene integrates multiple mechanisms including physical enrichment, chemical complexation and photocatalytic degradation. It enables the integrated remediation of mixed pollutants in water and soil, and overcomes the limitations of single-component materials.

## 6. Challenges and Future Perspectives

### 6.1. Current Challenges

Although carbon-based nanomaterials exhibit excellent structural superiority and application potential in agricultural and food safety detection as well as environmental pollution remediation, most current carbon-based sensing and adsorption systems remain confined to laboratory fundamental research. Their large-scale industrial implementation and practical commercialization still face multiple technical bottlenecks, mainly regarding complex matrix anti-interference capability, material stability and batch reproducibility, green scalable preparation, multifunctional integration, and industrial transformation efficiency.

Complex sample matrix interference is a prominent problem. Practical food and agricultural water systems contain complicated components, including proteins, lipids, natural pigments, organic acids, suspended particles and other interfering substances. These impurities are readily adsorbed on the surface of carbon-based nanomaterials, occupying active sensing and adsorption sites and interfering with interfacial electron transfer and optical signal responses. Such matrix effects inevitably reduce the detection accuracy and anti-interference ability of sensors and weaken the pollutant removal efficiency of adsorbents, severely restricting the practical application of carbon-based materials in complex real environmental and food matrices.

Material stability and batch reproducibility require further improvement. Certain carbon-based nanomaterials suffer from inherent structural defects. CNTs tend to agglomerate and stack during preparation and application, leading to the loss of numerous active sites. As emerging two-dimensional carbon materials, MXenes are vulnerable to oxidative degradation under high-temperature, high-humidity, and strong acidic and alkaline conditions, resulting in structural collapse and performance attenuation in sensing and adsorption. Furthermore, the insufficient controllability of existing preparation techniques causes evident differences in morphology, pore structure, particle size distribution and surface functional groups among different batches, leading to poor repeatability in sensing sensitivity and adsorption capacity, which fails to meet the requirements of standardized detection and large-scale engineering applications.

Scalable green preparation and cost constraints severely restrict industrialization. High-performance carbon-based nanomaterials such as high-quality graphene, MXenes and their MAX-phase precursors require complicated preparation procedures, rigorous reaction conditions and high-cost raw materials. In addition, several synthetic processes rely on toxic organic solvents and highly corrosive reagents, which pose risks of secondary environmental pollution and hinder low-cost, green and large-scale production. These limitations greatly restrict the industrial promotion and commercialization of carbon-based functional materials.

Insufficient multifunctional integration of intelligent systems: Most existing studies focus merely on single sensing detection or single adsorption purification functions, which can only realize qualitative and quantitative analysis or simple pollutant removal. Integrated intelligent systems that simultaneously achieve rapid detection, efficient enrichment and in situ catalytic degradation are still insufficient. The lack of full-cycle closed-loop regulation strategies for pollutant identification, enrichment and harmless treatment cannot satisfy the diversified demands of comprehensive agricultural pollution prevention and control.

Difficulties in practical transformation and industrial application: At present, most carbon-based sensing and remediation systems are only verified under ideal laboratory conditions. Systematic evaluation and long-term stability tests in practical scenarios, including food processing, storage and circulation, as well as field agricultural water and soil environments, are still lacking. The development of commercial portable detection equipment and modular environmental remediation devices based on carbon-based nanomaterials lags behind, resulting in a disconnection between fundamental research and practical application and slowing down the industrial transformation process.

In addition, the lack of systematic evaluations of the biological toxicity, environmental accumulation risks and food contact safety of carbon-based nanomaterials constitutes a critical bottleneck restricting their practical applications in agricultural food safety. Toxicological studies have proven that small-sized carbon nanotubes, graphene oxide, Maxine and other nanomaterials may enter organisms via skin contact, dietary intake or farmland runoff. These substances tend to accumulate in the liver, kidneys and plant root systems, triggering cellular oxidative stress and cell membrane damage. High-dose exposure can further suppress the proliferation of animal and plant cells. Once released into farmland soil and water environments, carbon-based nanomaterials are difficult to degrade naturally. Their long-term accumulation may alter the community structure of soil microorganisms and disrupt soil nutrient cycling, thereby posing potential ecological risks to farmland ecosystems. Moreover, when carbon-based nanomaterials are adopted as sensing probes or solid-phase extraction adsorbents that come into direct contact with food, trace nanoparticles may leach out and migrate into agricultural products. If the leached carbon nanoparticles enter the human body through daily diet, there is still no unified conclusion regarding their chronic toxic effects and cumulative toxicity after long-term exposure.

More importantly, no special safety regulations or limit standards have been formulated worldwide for carbon-based nanomaterials applied in food detection and agricultural environmental remediation. National food safety standards and agricultural product quality specifications in China only specify limit values for conventional heavy metals, pesticides and food additives, without defining the migration limits of various carbon-based nanomaterials upon food contact or their emission thresholds in farmland environments. Current nanomaterial regulatory frameworks in the European Union and the United States mainly target cosmetics and biomedicine, with no dedicated control rules for agricultural and food scenarios. The insufficiency of toxicological safety data and the absence of supporting standards make it impossible to complete food contact safety risk assessments for carbon-based sensing and remediation materials. This greatly impedes the commercial approval of carbon nanomaterial-based testing devices and farmland remediation agents, and severely limits the transition of such materials from laboratory research to practical agricultural food safety supervision.

### 6.2. Future Perspectives

In view of the existing bottlenecks of carbon-based nanomaterials in sensing detection and environmental remediation, combined with the development demands of smart agriculture, precise food safety supervision and green agricultural soil and water remediation, future research can focus on multifunctional integrated system construction, intelligent portable technology development, green low-carbon synthesis, multi-mode synergistic sensing and interdisciplinary innovation. These efforts will promote the transformation of carbon-based nanomaterial technology from laboratory exploration to large-scale, industrial and intelligent practical application.

Construction of multifunctional integrated intelligent platforms: Taking full advantage of the designable structure and tunable functions of carbon-based nanomaterials, novel composite functional materials and integrated systems that integrate rapid pollutant detection, efficient adsorption enrichment and in situ catalytic degradation should be developed. Breaking the limitations of single-function application, the integrated systems can realize full-cycle management of pollutant identification, enrichment and harmless degradation, and comprehensively improve the efficiency of agricultural and food pollution prevention as well as water and soil remediation.

Promotion of intelligent and portable detection technology: Combining smartphone imaging analysis, microfluidic chips, flexible sensing and intelligent signal processing technologies, traditional carbon-based sensing systems can be optimized into lightweight, miniaturized and integrated devices. The development of low-cost, user-friendly and field-deployable portable detection equipment can break the scenario restrictions of large precision instruments and laboratory detection. It enables the extensive application of high-performance carbon-based sensing technology in food production, market sampling, storage and circulation, as well as real-time agricultural environmental monitoring, facilitating on-site food safety supervision and dynamic agricultural environmental assessment.

Development of green synthesis technology based on agricultural and forestry wastes: Agricultural and forestry wastes such as bagasse, sisal fiber, chestnut shell and bamboo fiber can be adopted as sustainable carbon sources. Simple, low-energy and pollution-free modification strategies should be explored for the large-scale preparation of biomass-derived carbon functional materials. This approach realizes the high-value utilization of agricultural wastes, significantly reduces material preparation costs, avoids secondary pollution from traditional chemical synthesis, and establishes a green, low-carbon and sustainable material manufacturing and application system.

Establishment of multi-mode synergistic sensing systems. Integrating the superiorities of fluorescence, colorimetric, electrochemical and SERS sensing technologies, multi-signal synergistic and cross-verified composite sensing platforms can be constructed. Such systems can effectively compensate for the defects of single detection modes, such as weak anti-interference ability and high detection error, and significantly improve the accuracy, stability and reliability of trace pollutant detection in complex food and agricultural matrices.

Furthermore, future research should strengthen interdisciplinary integration of material science, analytical chemistry, agricultural engineering, food science and intelligent sensing technology. Continuous optimization of structural design and functional modification of carbon-based materials is essential to solve core problems including material agglomeration, oxidative deterioration, poor stability and insufficient batch reproducibility. The large-scale and intelligent application of carbon-based nanomaterials in food safety monitoring and agricultural environmental remediation will be comprehensively promoted, providing solid theoretical and technical support for ensuring food quality and safety, protecting agricultural ecological environments and realizing the green and sustainable development of modern agriculture.

To address the biological toxicity, environmental accumulation risks and regulatory gaps associated with carbon-based nanomaterials, systematic multi-level toxicological and ecological risk assessments should be carried out in follow-up research. Multi-stage toxicity evaluation models covering cell lines, model animals and plants, and farmland soil microorganisms need to be constructed to quantify dietary exposure doses and soil accumulation thresholds of carbon-based materials with different particle sizes and modification methods, and to identify low-toxicity and biodegradable modification strategies. Biocompatible modified carbon-based materials (including biomass carbon and heteroatom-doped low-toxicity carbon quantum dots) should be preferentially developed to reduce the risks of nanoparticle leaching and biological accumulation. Meanwhile, standardization committees for food, agriculture and nanomaterials should collaborate to formulate special standards tailored to agricultural product detection and farmland remediation scenarios. These standards shall specify the migration limits of carbon-based nanomaterials in contact with food and their discharge limits in farmland water, and formulate specifications governing the safe production, application and waste disposal of carbon-based sensing devices and remediation reagents. In addition, domestic approval procedures for nanotechnology applications in agricultural products should be refined by referencing international nanomaterial safety management frameworks, so as to fill the gaps in safety regulations for nanomaterials in the agricultural and food sectors and deliver comprehensive safety and institutional support for the industrialization of carbon-based nanomaterials.

## 7. Conclusions

In summary, graphene, CNTs, CQDs, BC, and emerging two-dimensional carbon-based materials possess unique physicochemical advantages, including large specific surface areas, excellent optoelectronic properties, facile functional tunability, and favorable biocompatibility, which endow them with broad application potential in agricultural and food safety research. When integrated with electrochemical sensing, fluorescence–colorimetric optical sensing and other advanced analytical techniques, carbon-based nanomaterials enable the highly sensitive and rapid screening of various hazardous contaminants in complex food matrices, such as heavy metal ions, pesticide residues, mycotoxins, antibiotics, and illegal additives. Furthermore, these materials support the non-destructive quality evaluation and real-time freshness monitoring of food products. In terms of agricultural environmental remediation, carbon-based nanomaterials achieve the effective enrichment, removal and harmless disposal of heavy metals and organic pollutants in farmland soil and water through high-efficiency physicochemical adsorption and electrocatalytic degradation. The excellent sensing and remediation performances of carbon-based materials are predominantly governed by three core mechanisms, namely optical signal regulation, electrochemical response modulation, and synergistic physicochemical adsorption and separation.

Despite the substantial progress made in the research of carbon-based nanomaterials, several critical bottlenecks still restrict their large-scale industrialization and practical on-site deployment. The main limitations include high fabrication costs, unsatisfactory structural stability and matrix anti-interference capability in complex practical environments, poor batch-to-batch reproducibility, and the insufficient development of multifunctional integrated systems. Future research priorities focus on the exploitation of low-cost, environmentally benign and sustainable biomass-based synthesis strategies. Continuous structural optimization and functional composite modification are essential to improve the matrix anti-interference ability and environmental adaptability of carbon-based materials. Combined with intelligent sensing technologies, these advancements can facilitate the miniaturization, portability and systematic integration of detection equipment. The breakthrough of existing technical barriers will accelerate the transformation of carbon-based nanomaterials from laboratory-scale fundamental research to practical field applications. This provides robust technical support for the precise quality detection of agricultural products and comprehensive ecological remediation of farmland environments, further promoting the green, safe and intelligent development of the modern agriculture and food industries.

## Figures and Tables

**Table 1:** Mechanisms, performance and application comparison of four carbon-based nanomaterial sensing systems.

Item	Electrochemical Sensing	Fluorescence Sensing	Colorimetric Nanozyme Sensing	SERS Sensing
Core Carbon-Based Materials	Graphene, multi-walled carbon nanotubes (MWCNTs), MXene	Carbon quantum dots (CQDs), graphene oxide (GO), g-C_3_N_4_ quantum dots	MWCNTs, graphene–noble metal composites	Graphene/MXene composite gold/silver substrates
Core Mechanism	1. Carbon skeleton accelerates interfacial electron transfer and reduces charge transfer resistance; 2. surface functional groups serve as stable anchors for aptamers/enzymes; 3. nanozyme-catalyzed amplification of redox currents	Fluorescence quenching/recovery modulated via FRET/PET electron transfer; heteroatom doping improves fluorescence quantum yield	Peroxidase-like activity of carbon-based nanozymes catalyzes chromogenic reactions of substrates	Target enrichment by carbon matrices coupled with plasmonic Raman signal amplification from noble metals
Signal Transduction Pathway	Current/potential voltammetric signals	Fluorescence intensity, fluorescence wavelength shift	Solution absorbance, naked-eye color transition	Intensity of Raman characteristic peaks
Advantages	Resistant to colored matrix interference, wide linear range, quantitative capability, compatible with miniaturized devices	Visualizable readout; dual-signal ratiometric calibration minimizes measurement bias	Fully instrument-free visualization, extremely low cost	Single-molecule ultrasensitivity, simultaneous multi-component detection
Inherent Limitations	No naked-eye readout, requires an electrochemical workstation	Susceptible to fluorescence quenching by food pigments and humic acids; severe matrix interference	Color readout vulnerable to turbidity interference; moderate quantitative accuracy	High instrumentation cost, poor batch-to-batch reproducibility, peak overlapping in complex matrices
Typical LOD Range	ng/L to pg/L level	μg/L to fg/mL level	nM level	ppb to ppt level
Target Analytes	Heavy metals, pesticides and veterinary drugs, mycotoxins	Heavy metals, mycotoxins, volatile amines in fresh produce	Veterinary drugs, illegal pesticide additives	Pesticides, trace organic pollutants (e.g., polychlorinated biphenyls)
Field Applicability	Portable electrodes enable on-site field deployment	Test strips/thin films support non-destructive in-package monitoring	Rapid screening test strips for on-site market inspection	Primarily applied for laboratory-based precision detection
References	[11,41,49,82,90,91,99,100,101]	[42,58,59,61,62,77,84,88,93]	[58,100]	[11,20]

**Table 2:** Mechanisms and performances of pollutant adsorption and degradation by typical carbon-based nanoremediation materials.

Carbon-Based Remediation Material	Dominant Adsorption Mechanism	Synergistic Effects	Typical Maximum Adsorption Capacity (mg/g)	Regeneration Performance	Applicable Pollutants	Key Limitations	References
Pristine Biochar	Physical adsorption (van der Waals forces within hierarchical pores)	Weak complexation by surface hydroxyl/carboxyl groups	Moderate (50–120 mg/g)	Fair (>30% capacity decay after five cycles)	Organic dyes, low-concentration heavy metals	Scarce active sites, poor target selectivity	[66,105]
Functionalized Modified Biochar (LDH/magnetic composite)	Chemical complexation, ion exchange	Magnetic separation, metal ion reduction	Cr(VI): 60 [44]; Pb^2+^: 204 [71];	Excellent (~60% capacity retained after 10 cycles)	Heavy metals (Cd^2+^/Pb^2+^, Cr(VI))	Complex preparation and modification workflows	[44,65,70,71]
β-Cyclodextrin (β-CD)-Modified MWCNTs	Host–guest inclusion (within cyclodextrin cavities)	Electrostatic adsorption on nanotube sidewalls	Moderate to high (100–200 mg/g)	Good (stable performance over eight cycles)	Organic pesticides, organic dyes	Nanotube aggregation readily blocks internal pore channels	[50]
MXene/g-C_3_N_4_ Composite Adsorbent	Electrostatic adsorption, surface complexation	In situ photocatalytic degradation of adsorbed pollutants	Ultrahigh (>300 mg/g)	Poor (prone to oxidative deactivation under humid conditions)	Heavy metal–organic combined pollution	High production cost, unsatisfactory storage stability	[11,74,79]
Superhydrophobic Carbon Aerogel	Physical adsorption (porous structure for oil retention)	Hydrophobic–oleophilic selective separation	Extremely high (70–144 g/g for oils/organic solvents)	Outstanding (>90% capacity retained after 10 cycles)	Oil spills, organic solvents	Only effective for aqueous organic phases; negligible heavy metal removal capacity	[97]

## Data Availability

No new data were created or analyzed in this study. Data sharing is not applicable to this article.
